# Saffron Processing Wastes as a Bioresource of High-Value Added Compounds: Development of a Green Extraction Process for Polyphenol Recovery Using a Natural Deep Eutectic Solvent

**DOI:** 10.3390/antiox8120586

**Published:** 2019-11-25

**Authors:** Achillia Lakka, Spyros Grigorakis, Ioanna Karageorgou, Georgia Batra, Olga Kaltsa, Eleni Bozinou, Stavros Lalas, Dimitris P. Makris

**Affiliations:** 1 School of Agricultural Sciences, University of Thessaly, N. Temponera Street, 43100 Karditsa, Greece; ACHLAKKA@uth.gr (A.L.); ikarageorgou@uth.gr (I.K.); gbatra@uth.gr (G.B.); okaltsa@uth.gr (O.K.); empozinou@uth.gr (E.B.); slalas@uth.gr (S.L.); 2 Food Quality & Chemistry of Natural Products, Mediterranean Agronomic Institute of Chania (M.A.I.Ch.), International Centre for Advanced Mediterranean Agronomic Studies (CIHEAM), P.O. Box 85, 73100 Chania, Greece; grigorakis@maich.gr

**Keywords:** anthocyanins, antioxidants, deep eutectic solvents, extraction, polyphenols, saffron

## Abstract

The current investigation was undertaken to examine saffron processing waste (SPW) as a bioresource, which could be valorized to produce extracts rich in antioxidant polyphenols, using a green, natural deep eutectic solvent (DES). Initially, there was an appraisal of the molar ratio of hydrogen bond donor/hydrogen bond acceptor in order to come up with the most efficient DES composed of L-lactic acid/glycine (5:1). The following step was the optimization of the extraction process using response surface methodology. The optimal conditions thus determined were a DES concentration of 55% (*w*/*v*), a liquid-to-solid ratio of 60 mL g^−1^, and a stirring speed of 800 rounds per minute. Under these conditions, the extraction yield in total polyphenols achieved was 132.43 ± 10.63 mg gallic acid equivalents per g of dry mass. The temperature assay performed within a range of 23 to 80 °C, suggested that extracts displayed maximum yield and antioxidant activity at 50–60 °C. Liquid chromatography-mass spectrometry analysis of the SPW extract obtained under optimal conditions showed that the predominant flavonol was kaempferol 3-*O*-sophoroside and the major anthocyanin delphinidin 3,5-di-*O*-glucoside. The results indicated that SPW extraction with the DES used is a green and efficient methodology and may afford extracts rich flavonols and anthocyanins, which are considered to be powerful antioxidants.

## 1. Introduction

In recent years, the agri-food sector has been acknowledged as a major contributor to the global environmental burden. Processing of plants (fruit, vegetables, tubers etc.) for the production of plant food commodities is considered to be a major concern, since a vast amount of waste material may be generated [1]. Plant processing waste is residual biomass rich in moisture and microbial loads and can be a direct risk associated with environmental pollution. On the other hand, an ever-increasing number of current studies on plant food processing residues suggests the presence of a wide range of bioactive compounds in different waste fractions. These bioactive substances are primarily secondary plant metabolites, belonging to polyphenols, carotenoids, essential oils, resins, etc. Therefore, plant food processing waste and residues are highly regarded as very promising sources of bioactive compounds, with applications in food technology, pharmaceuticals, and cosmetics [2,3].

To date, the development of methodologies for high-performance and time-effective extraction of polyphenols from plant matrices is a challenge, because of the inherent limitations of conventional extraction methods. The valorization of polyphenols as bioactive ingredients at various commercial levels has shifted research to low-cost, eco-friendly, and efficient extraction techniques, based on a green philosophy [4]. A basic concept of such an approach would be the use of novel, green solvents, which would be devoid of the disadvantages that characterize the conventional, volatile, petroleum-based solvents. In this view, the emerging liquids known as deep eutectic solvents (DES) would appear to be solid ground for the implementation of green processes for the production of polyphenol-enriched extracts.

DES are novel materials, which can be synthesized using natural substances, such as sugars, polyols, organic acids and their salts, amino acids, etc. [5]. They are usually composed of a hydrogen bond donor (HBD) and a hydrogen bond acceptor (HBA). The ongoing research on these solvents has provided substantial evidence that they can be highly effective in polyphenol extraction, surpassing the potency of conventional solvents, such as methanol. On the other hand, DES are not volatile, their production does not depend on fossil sources, and they have very attractive characteristics, including tunability of composition (and thus regulation of their properties), lack of toxicity, recyclability, and low cost. It is not surprising, therefore, that over the past five years, numerous DES have been synthesized and tested for their potency to extract polyphenolic compounds [6,7].

The plant *Crocus sativus* (Iridaceae), known widely as saffron, is a perennial herb that has been acknowledged since antiquity for its culinary uses and medicinal properties [8]. The most precious part of the plant is the stigmas, which are collected and dried to produce the world’s most expensive spice. Following screening and separation, the rest of the flower, composed essentially of the tepals (undifferentiated petals and sepals), is rejected as a residual material. However, emerging evidence has showed that saffron petals contain an array of bioactive polyphenols, including a series of flavonol glycosides and anthocyanin pigments. Several of these constituents were reported to possess multiple beneficial bioactivities [9], and on this evidence, a few extraction methodologies were developed, with the aim of producing polyphenol-containing extracts from saffron processing waste (SPW) [10,11,12,13].

However, to the best of the authors’ knowledge, the use of DES has never been reported for SPW extraction. The present investigation describes the development of a green extraction methodology for the effective recovery of SPW polyphenols, using a DES composed of L-lactic acid (HBD) and glycine (HBA). The study included the synthesis of the most efficient system by screening a range of HBD:HBA molar ratios and then the optimization by deploying response surface methodology and a temperature assay. The polyphenolic composition of the optimally obtained extract was assessed by performing liquid chromatography, mass spectrometry analyses.

## 2. Materials and Methods

### 2.1. Chemicals

Glycine (99.5%) was from Applichem (Darmstadt, Germany). Iron chloride hexahydrate was from Merck (Darmstadt, Germany). Rutin (quercetin 3-*O*-rutinoside) hydrate, kaempferol 3-*O*-glucoside, 2,4,6-tris(2-pyridyl)-*s*-triazine (TPTZ), 2,2-diphenylpicrylhydrazyl (DPPH) and Folin-Ciocalteu reagent were from Sigma-Aldrich (St. Louis, MO, USA). L-Lactic acid, sodium carbonate anhydrous (99%), ascorbic acid (99.5%), and sodium acetate trihydrate and aluminium chloride anhydrous (98%) were from Penta (Praha, Czechia). Gallic acid hydrate was from Panreac (Barcelona, Spain). Pelargonin (pelargonidin 3,5-di-*O*-glucoside) chloride was from Extrasynthese (Genay, France).

### 2.2. Plant Material and Handling

Saffron (*Crocus sativus* L.) processing waste (SPW), composed essentially of saffron tepals, was collected immediately after manual processing of saffron flowers from a processing plant located in Kozani (West Macedonia, Greece). The plant material was transferred to the laboratory within 24 h and dried at 55 °C for 48 h in a laboratory oven (Binder BD56, Bohemia, NY, USA). Dried SPW was pulverized in a ball-mill to give powders with approximate average particle diameter of 0.317 mm, and stored in air-tight vessels at −18 °C until used.

### 2.3. DES Synthesis

Synthesis of the DES used in this study was based on a previous protocol [14]. Exact weights of L-lactic acid (HBD) and glycine (HBA) were transferred into a round-bottom glass flask and heated moderately (75–80 °C) for approximately 120 min until the formation of a perfectly transparent liquid. Heating was provided by an oil bath placed on a thermostat-equipped hotplate (Witeg, Wertheim, Germany). The liquid was allowed to acquire room temperature and stored in a sealed vial, in the dark. Inspection for appearance of crystals that would indicate instability was performed at regular intervals over six weeks.

### 2.4. Batch Stirred-Tank Extraction

Exact mass of 0.570 g of dried plant material was introduced into a 50-mL round-bottom flask with 20 mL of solvent to give a liquid-to-solid ratio (R_L/S_) of 35 mL g^−1^. The flask was immersed into oil bath and heated by means of a thermostat-equipped hotplate. Extractions were carried out for 150 min, at 50 °C, under magnetic stirring set at 500 rpm. All DES were tested as 70% (*w*/*v*) aqueous mixtures. Extractions with deionised water, 60% (*v*/*v*) aqueous ethanol and 60% (*v*/*v*) aqueous methanol were used as control. After the extraction, samples were centrifuged at 10,000× *g* for 10 min and the supernatant was used for all analyses.

### 2.5. Extraction Optimization with Response Surface Methodology (RSM)

The scope of RSM was the implementation of a mathematical model to predict polyphenol extraction performance from SPW using the most efficient DES synthesized. The mode chosen was a Box-Behnken experimental design with three central points. Key extraction variables including the concentration of DES in aqueous mixtures (*C*_DES_), the liquid-to-solid ratio (R_L/S_) and the stirring speed (S_S_) [15] were taken into account and termed X_1_, X_2_, and X_3_, respectively (Table 1). Yield in total polyphenols (Y_TP_) was the screening response and the three independent variables were coded between −1 (lower limit) and 1 (upper limit). Codification was performed with the following equation [16]:

(1) $$X_{i}\;=\;(\frac{z_{i}-\;z_{1}^{0}}{\Delta z_{i}})\;\times \;\beta_{d}.$$

Δ*z_i_* is the distance between the real value at the central design point and the real value in the upper or lower limit of a variable; *β*_d_ is the major coded limit value in the matrix for each variable, and *z*^0^ is the real value at the central point. The equation (mathematical model) obtained by fitting the function to the experimental data was evaluated by ANOVA. Visual model representation was done by 3D surface response plots.

### 2.6. Total Polyphenol Determination

An established methodology was used [17]. Samples were diluted 1:50 with 0.5% aqueous formic acid prior to determinations. A volume of 0.1 mL of diluted sample was transferred into a 1.5-mL Eppendorf tube and mixed with 0.1 mL Folin–Ciocalteu reagent. The mixture was allowed to react for 2 min and then 0.8 mL of sodium carbonate (5% *w*/*v*) was added, followed by 20-min incubation at 40 °C, in a water bath. After incubation, the absorbance at 740 nm was read and total polyphenol concentration (*C*_TP_) was determined from a calibration curve constructed with gallic acid (10–80 mg L^−1^). Extraction yield in total polyphenols was expressed as mg gallic acid equivalents (GAE) per g dry mass (dm).

### 2.7. Total Flavonoid Determination

For total flavonoids, a previously published protocol was employed [18]. Volume of 0.1 mL of appropriately diluted sample was combined with 0.86 mL 35% (*v*/*v*) aqueous ethanol and 0.04 mL of reagent consisted of 5% (*w*/*v*) AlCl_3_ and 0.5 M CH_3_COONa. After 30 min at room temperature the absorbance was obtained at 415 nm. Total flavonoid concentration (*C*_TFn_) was calculated from a calibration curve using rutin as standard (15–300 mg L^−1^). Yield in total flavonoids (Y_TFn_) was estimated as mg rutin equivalents (RtE) per g dm.

### 2.8. Determination of the Antiradical Activity (A_AR_)

The determination was based on the stable radical probe DPPH using a stoichiometric assay [19]. All samples were diluted 1:50 with methanol just before the analysis, and 0.025 mL of sample was mixed with 0.975 mL DPPH (100 μM in methanol) at room temperature. Absorbance readings at 515 nm were performed at *t* = 0 min (immediately after mixing) and at *t* = 30 min. The A_AR_ of the extract was then computed as follows:

(2) $$A_{\mathrm{AR}}\;=\;\frac{C_{\mathrm{DPPH}}}{C_{\mathrm{TP}}}\times \left(1-\frac{A_{515\left(f\right)}}{A_{515\left(i\right)}}\right)\times Y_{\mathrm{TP}}.$$

*C*_DPPH_ and *C*_TP_ are the DPPH concentration (μM) and total polyphenol concentration (mg L^−1^) in the reaction mixture, respectively. A_515(f)_ corresponds to A_515_ at *t* = 30 min and A_515(i)_ to A_515_ at *t* = 0. Y_TP_ is the extraction yield (mg g^−1^) in TP of each of the extracts tested. A_AR_ was calculated as μmol DPPH g^−1^ dm.

### 2.9. Determination of the Reducing Power (P_R_)

The ferric-reducing power assay was performed as previously described [19]. Before the analysis, samples were diluted 1:50. Then, 0.05 mL of the sample was incubated with 0.05 mL FeCl_3_ (4 mM in 0.05 M HCl) at 37 °C in a water bath for 30 min. Following incubation, 0.9 mL of TPTZ solution (1 mM in 0.05 M HCl) was added, and the mixture was allowed to stand at room temperature for further 5 min. The absorbance was obtained at 620 nm and P_R_ was reported as μmol ascorbic acid equivalents (AAE) g^−1^ dm using an ascorbic acid calibration curve (50–300 μM).

### 2.10. Liquid Chromatography Diode Array Mass Spectrometry (LC-DAD-MS)

A modification of a method reported elsewhere was used [20]. The apparatus was a Finnigan (San Jose, CA, USA) MAT Spectra System P4000 pump, a UV6000LP diode array detector, and a Finnigan AQA mass spectrometer. Analyses were performed with a Fortis RP-18 column, 150 mm × 2.1 mm, 3 μm, at 40 °C, with a 10-μL injection loop. Acquisition of mass spectra at 20 and 70 eV was performed with electrospray ionization (ESI) in positive ion mode, using the following settings: probe temperature was 250 °C, source voltage at 25 V, detector voltage at 450 V, and capillary voltage at 4 kV. The eluents were (A) 2% acetic acid and (B) methanol and the flow rate was 0.3 mL min^−1^. Elution was carried out as follows: 0–30 min, 0–100% methanol; 30–40 min, 100% methanol.

### 2.11. High-Performance Liquid Chromatography Diode Array (HPLC-DAD)

The analysis was carried out on a Shimadzu CBM-20A liquid chromatograph (Shimadzu Europa GmbH, Duisburg, Germany) equipped with an SIL-20AC auto sampler and a CTO-20AC column oven. Detection was carried out using a Shimadzu SPD-M20A detector. The system was interfaced by Shimadzu LC solution software. Chromatography was carried out on a Phenomenex Luna C18(2) column (100 Å, 5 μm, 4.6 × 250 mm) (Phenomenex, Inc., Torrance, CA, USA). Columns were maintained at a temperature of 40 °C. Eluents were (A) 0.5% aqueous formic acid and (B) 0.5% formic acid in MeCN/water (6:4), and the flow rate was 1 mL min^−1^. A 20 μL sample was injected into the high-performance liquid chromatography (HPLC). Following is the elution program used: 100% A to 60% A in 40 min, 60% A to 50% A in 10 min, 50% A to 30% A in 10 min, and then isocratic elution for another 10 min. The column was washed with 100% MeCN and re-equilibrated with 100% eluent A before the next injection. Quantification was performed with calibration curves (0–50 μg mL^−1^) constructed with kaempferol 3-*O*-glucoside (R^2^ = 0.9999), rutin (R^2^ = 0.9990), and pelargonin (R^2^ = 0.9999).

### 2.12. Statistical Analysis

Extractions were repeated at least twice, and all determinations were carried out in triplicate. Values presented are means ± standard deviation (sd). Linear regression analysis was used to establish linear correlations, at least at a 95% significance level (*p* < 0.05), using SigmaPlot™ 12.5 (Systat Software Inc., San Jose, CA, USA). The design of experiment, response surface methodology and all associated statistics were performed with JMP™ Pro 13 (SAS, Cary, NC, USA).

## 3. Results and Discussion

### 3.1. DES Synthesis and the Effect of HBD:HBA Molar Ratio ($R_{mol}^{D/A}$)

**Figure 1:**
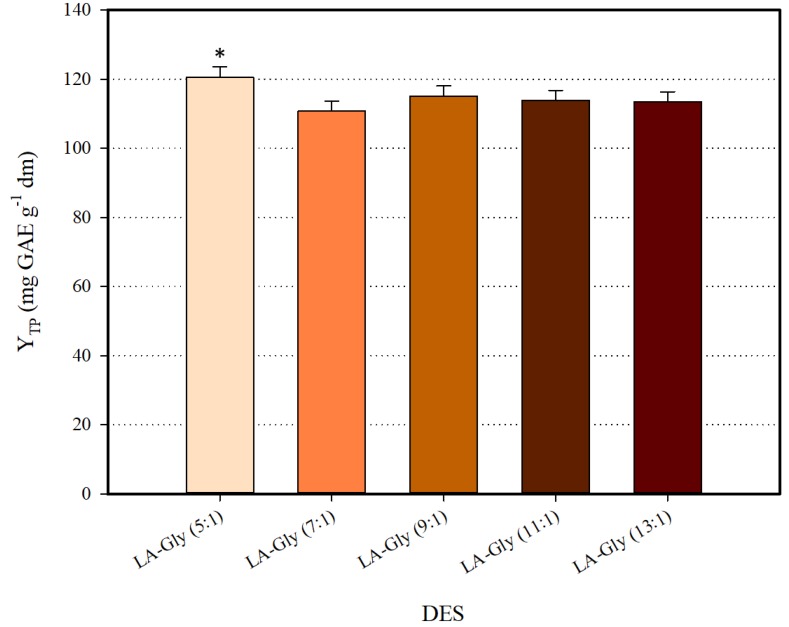
Screening of HBD:HBA ratio for the selection of the most efficient deep eutectic solvent (DES). Asterisk (*) denotes statistically different value (*p* < 0.05).

The first report on L-lactic acid (LA) and glycine (Gly) combination pointed out that stable DES may be formed at $R_{\mathrm{mol}}^{D/A}$ > 3 [21]. In latter studies, DES composed of LA and Gly exhibited stability at $R_{\mathrm{mol}}^{D/A}$ ≥ 5, and it was also demonstrated that $R_{\mathrm{mol}}^{D/A}$ may significantly affect DES efficiency in extracting phenolics [22]. On such a basis, synthesis and screening of a series of LA-Gly DES with $R_{\mathrm{mol}}^{D/A}$ ranging from five to 13, was the first stage in the development of an efficient solvent. All DES synthesized were tested for polyphenol recovery as 70% (*w*/*v*) aqueous mixtures. Screening results are depicted in Figure 1. The DES LA-Gly (5:1) was proven to be the highest-performing system, providing significantly increased Y_TP_ (*p* < 0.05). This finding evidenced the potency of LA-Gly (5:1) for polyphenol recovery, and on this ground, this DES was chosen for all further processes.

### 3.2. Assessment of the DES Extraction Efficiency

To better illustrate the efficiency of LA-Gly (5:1), an appraisal was carried out by comparing the DES performance with that of two other green solvents, namely 60% (*v*/*v*) aqueous ethanol and water. Extractions with a commonly used solvent, 60% (*v*/*v*) aqueous methanol, were also performed. For the appraisal, in addition to Y_TP_, the Y_TFn_, A_AR_ and P_R_ were also considered, and the results are analytically displayed in Table 2. LA-Gly (5:1) gave higher Y_TP_, which was statistically significant (*p* < 0.05). Regarding Y_TFn_ and P_R_, extraction of SPW with LA-Gly (5:1) also afforded higher but statistically non-significant values, whereas A_AR_ of the LA-Gly (5:1) was lower compared to the extracts obtained with the control solvents. Based on these results, it was deemed that LA-Gly (5:1) was indeed the highest-performic system.

### 3.3. Optimisation of Extraction Performance

Response surface methodology was deployed to assess the effect of three basic extraction variables (*C*_DES_, R_L/S_, S_S_) on the performance of LA-Gly (5:1) to recover polyphenolic antioxidants. The objective was the fit of polynomial equations (models) to the experimental data, in order to describe effectively the behavior of the data set for making statistical previsions. Assessment of the fitted models was based on the ANOVA (Table 3). By neglecting the non-significant terms, the first-degree equation (mathematical model) was

Y_TP_ = 115.32 + 3.24X_1_ + 8.67X_2_ + 5.73X_3_ − 5.11X_1_X_2_ − 4.93X_1_X_3_. (3)

The square correlations coefficient (R^2^) and the *p*-value are indicators of the total variability around the mean calculated by the model. Measured and predicted Y_TP_ values for each material extracted and for each design point are analytically presented in Table 4. Because total R^2^ of the model was 0.95, and the *p* value (assuming a confidence interval of 95%) was highly significant (0.0080) (*F* value for lack-of-fit = 30.1016), Equation (3) showed excellent fitting to the experimental data.

**Figure 2:**
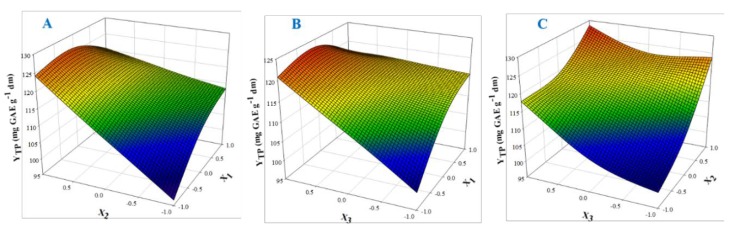
Three-dimensional plots displaying the effect of process (independent) variables on the total polyphenol yield (YTP). For variable assignment, see Table 1. Plots (**A**), (**B**) and (**C**) show covariation of variables X1 and X2, X1 and X3, and X2 and X3, respectively.

**Figure 3:**
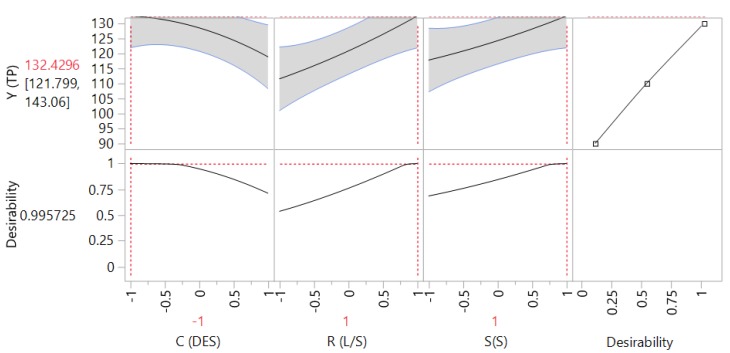
Desirability function showing the maximum predicted response upon setting process (independent) variable values at the predicted optima.

The 3D plots created based on the model are given in Figure 2, to readily portray the effect of the process variables on the response (Y_TP_). The desirability function enabled the simultaneous optimization of the levels of all three variables in order to attain the best system performance (Figure 3), and the sets of conditions to achieve the highest theoretical yield were estimated to be *C*_DES_ = 55% (*w*/*v*), R_L/S_ = 60 mL g^−1^ and S_S_ = 800 rpm. Under these conditions, the maximum theoretical Y_TP_ was 132.43 ± 10.63 mg GAE g^−1^ dm. To confirm the validity of the model, three extractions of each material were carried out under the optimal conditions. The Y_TP_ determined was 128.00 ± 1.94 mg GAE g^−1^ dm, suggesting that the theoretical optimum settings for *C*_DES_, R_L/S_, and S_S_ may be applied with high reliability.

Judging by the model, *C*_DES_ (X_1_) had a direct positive influence on Y_TP_, and the same was seen for R_L/S_ (X_2_). Likewise, S_S_ (X_3_) had a positive impact on SPW polyphenol extraction. On the other hand, cross terms *C*_DES_ (X_1_) and R_L/S_ (X_2_), and *C*_DES_ (X_1_) and S_S_ (X_3_) had a negative effect on Y_TP_. The predicted *C*_DES_ levels implied the use of a significantly higher water amount compared with previous results from polyphenol extraction with DES, which indicated that 80% (*w*/*w*) to be the most appropriate *C*_DES_ for high extraction yield [23,24,25,26]. Suitable DES mixing with water is indispensable for regulation of properties crucial to solid-liquid extraction, such as viscosity and polarity [27]. However, *C*_DES_ cannot be below a certain level because excessive water amount would cause DES decomposition and therefore the intrinsic DES properties would be abolished [28].

Variable R_L/S_ is a strongly influential factor regarding solid–liquid extraction, as it affects concentration gradient between the solid particles and the liquid phase, which is the driving force for the manifestation of diffusion phenomena. Recently, it was demonstrated that raising R_L/S_ from 10 to 50 mL g^−1^ may significantly increase diffusivity [29]. Conventional solvent extraction may require R_L/S_ as high as 120 mL g^−1^ [30,31], but for polyphenol extractions with DES, lower R_L/S_ levels ranging from 29–50 mL g^−1^ are usually effective [32,33,34]. The optimal R_L/S_ estimated was 60 mL g^−1^, indicating that higher concentration gradients may be necessary for effective polyphenol leaching into the liquid phase.

In a similar manner, S_S_ was shown to play an important role in solid–liquid extraction, and appropriate S_S_ regulation may give significantly higher extraction yields [29,31]. Sufficiently high S_S_ causes turbulence in the extraction tank, and this in turn may increase mass transfer rate. In this line, S_S_ has been demonstrated to provide increased polyphenol diffusivity [29]. On the other hand, high S_S_ may lead to incomplete diffusivity because higher turbulence could shift the equilibrium toward polyphenol adsorption rather than diffusion. At this point, characteristics such as the viscosity of the liquid phase (solvent), which is tightly associated with R_L/S_, should also be considered. Such a hypothesis might explain the combined effect observed between S_S_ and *C*_DES_ (cross term X_1_X_3_) for SPW polyphenol extraction.

### 3.4. Temperature Effects

**Figure 4:**
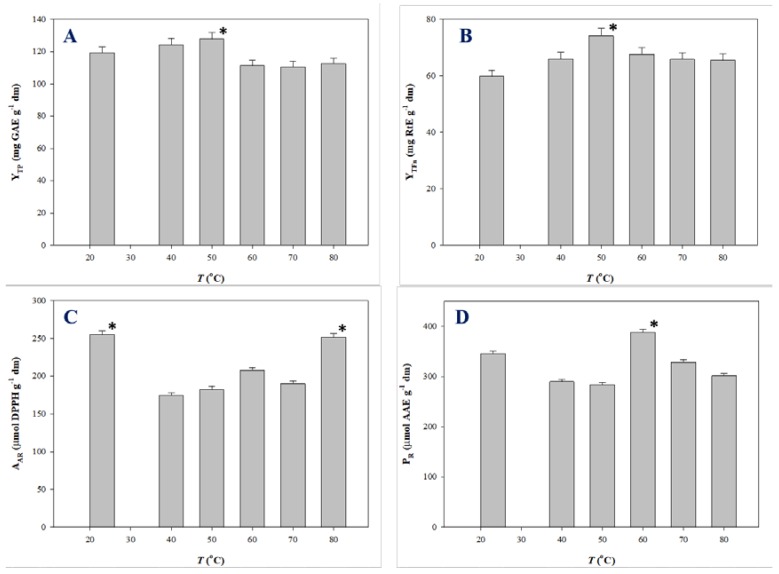
Variation of Y_TP_ (**A**), Y_TFn_ (**B**), A_AR_ (**C**), and P_R_ (**D**) as a function of *T*. Bars indicate standard deviation. Asterisk (*) denotes statistically different value (*p* < 0.05).

Polyphenols are thermosensitive molecules and in several cases temperature increase does not generate a monotonous effect on the extraction yield and antioxidant activity. Such a behavior was demonstrated for the extraction of onion solid waste [35,36], red grape pomace [37], and *Moringa oleifera* leaves [25]. Therefore, the impact of temperature on the production of polyphenol-enriched extracts with improved antioxidant characteristics merits thorough investigation. For this reason, extractions under optimal conditions were carried out at temperatures varying from 23 (ambient temperature) to 80 °C, and the extracts produced were assessed by determining Y_TP_, Y_TFn_, A_AR_, and P_R_. The outcome of this assay is analytically presented in Figure 4.

Y_TP_ for SPW extraction peaked at 50 °C (128.03 mg GAE g^−1^ dm), while a decline was recorded thereafter (Figure 4A). Likewise, maximum Y_TFn_ was found at 50 °C (Figure 4B). A_AR_ exhibited fluctuations within a narrow range, the highest A_AR_ being at 23 °C (255.15 μmol DPPH g^−1^ dm) (Figure 4C). Significant differentiation was seen for the evolution of P_R_, which gave maximum levels at 60 °C (388.15 μmol AAE g^−1^ dm) (Figure 4D) Considering all the above parameters, the data obtained suggested that SPW extraction provided polyphenol-enriched extracts with enhanced antioxidant activity at around 50–60 °C. This finding may be evidence of the thermal stability of SPW constituents.

### 3.5. Polyphenolic Composition

**Figure 5:**
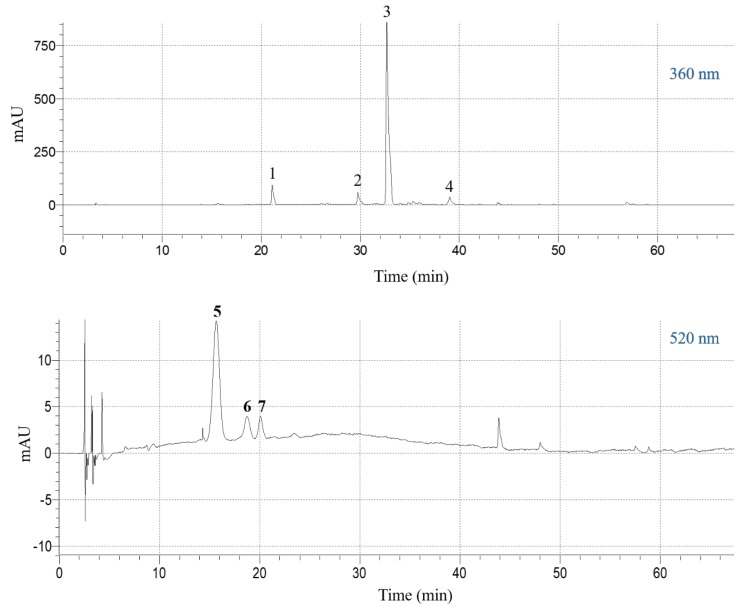
High-performance liquid chromatography (HPLC) traces of the SPW extract obtained with the DES, under optimal conditions (*C*_DES_ = 55% (*w*/*v*), R_L/S_ = 60 mL g^−1^, S_S_ = 800 rpm, *T* = 50 °C). The upper and lower traces were monitored at 360 and 520 nm, respectively. Peak assignment: 1, kaempferol 3-*O*-sophoroside 7-*O*-glucoside; 2, quercetin 3-*O*-sophoroside; 3, kaempferol 3-*O*-sophoroside; 4, kaempferol 3-*O*-glucoside; 5, delphinidin 3,5-di-*O*-glucoside; 6, petunidin 3,5-di-*O*-glucoside; 7, delphinidin 3-*O*-glucoside.

The extract obtained under optimal conditions (*C*_DES_ = 55% (*w*/*v*), R_L/S_ = 60 mL g^−1^, S_S_ = 800 rpm, *T* = 50 °C) was analyzed by liquid chromatography-diode array-mass spectrometry, to detect and tentatively identify the major polyphenolic constituents. The compound with retention time (Rt) 15.62 min (peak #5) in the chromatogram monitored at 520 nm (Figure 5), gave a molecular ion at *m*/*z* = 627 and a diagnostic fragment at *m*/*z* = 465. This peak was identified as delphinidin 3,5-di-*O*-glucoside (Table 5). Likewise, the peak at 18.72 min (peak #6) yielded a molecular ion at *m*/*z* = 641 and a characteristic fragment at *m*/*z* = 465, and it was assigned to petunidin 3,5-di-*O*-glucoside. Finally, the peak with Rt 20.08 min (peak #7) was identified as delphinidin 3-*O*-glucoside, based on its major peak (*m*/*z* = 465) and its fragment at *m*/*z* = 303 [38].

The chromatogram at 360 nm revealed the existence of four principal constituents (Figure 5). Peak #1 yielded a pseudo-molecular ion at *m*/*z* = 773 and three diagnostic fragments at *m*/*z* = 611 (loss of glucose) at *m*/*z* = 449 (loss of sophorose) and at *m*/*z* = 287 (aglycone). This compound was tentatively identified as kaempferol 3-*O*-sophoroside 7-*O*-glucoside (Table 5). Similarly, peak #2 gave a pseudo-molecular ion at *m*/*z* = 627 and the aglycone ion at *m*/*z* = 303 and it was assigned to quercetin 3-*O*-sophoroside. Peak #3 displayed a pseudo-molecular ion at *m*/*z* = 611 and fragments at *m*/*z* = 449 (loss of glucose) and *m*/*z* = 287 (aglycone), and its structure was assigned to kaempferol 3,7-di-*O*-glucoside. Peak #4 showed pseudo-molecular and fragment ions at *m*/*z* = 449 and 287, respectively, and it was identified as kaempferol 3-*O*-glucoside [39].

On the ground of the quantitative data presented in Table 6, the flavonol composition of the extract was characterized by relatively high amounts of kaempferol 3-*O*-sophoroside (36.43 ± 2.55 mg g^−1^ dm), accompanied by much lower proportions of kaempferol 3-*O*-sophoroside 7-*O*-glucoside and quercetin 3-*O*-sophoroside. Regarding anthocyanins, the profile was dominated by delphinidin 3,5-di-*O*-glucoside (6.28 ± 0.44 mg g^−1^ dm). This outcome is in accordance with previous studies [40,41], which showed that the predominant flavonol found in aqueous SPW extract was kaempferol 3-*O*-sophoroside (30.34 mg g^−1^ dm), followed by kaempferol 3-*O*-sophoroside 7-*O*-glucoside (5.6 mg g^−1^ dm) and quercetin 3-*O*-sophoroside (4.01 mg g^−1^ dm). However, important amounts of delphinidin 3,5-di-*O*-glucoside (23.19 mg g^−1^ dm) were determined in ethanolic extract, whereas the aqueous extract was relatively rich in petunidin 3,5-diglucoside (3.97 mg g^−1^ dm). Data from another investigation were in line, giving values of 12.60 and 3.94 mg g^−1^ dm, for kaempferol 3-*O*-sophoroside and delphinidin 3,5-di-*O*-glucoside, respectively, in water/methanol extracts [38]. Although the polyphenolic composition of SPW could be influenced by the genetic background (variety), the area of origin and sample processing conditions, comparison of the total polyphenol content determined in this study (Table 6) with the values reported in the literature would indicate that SPW extraction with the DES used, under the optimal conditions estimated, an efficient process to produce polyphenol-enriched extracts with important antioxidant activity.

## 4. Conclusions

Saffron processing waste was used as raw material for the recovery of antioxidant polyphenols using a natural deep eutectic solvent composed of L-lactic acid and glycine. It was demonstrated that the HBD:HBA molar ratio can significantly affect extraction yield, hence initial screening of the most appropriate HBD:HBA molar ratio should be a key step in the development of similar processes. Furthermore, the response surface optimization of polyphenol extraction from SPW clearly showed that the proportion of solvent/water, as well as the liquid-to-solid ratio and the stirring speed may crucially affect the extraction performance. Therefore, these variables are to be suitably adjusted in order to maximize extraction yield. Likewise, temperatures higher than 50–60 °C were shown to have a negative impact on the extraction yield, and this is another salient process parameter that should be taken into consideration. SPW extraction under the optimally defined conditions gave extracts rich in polyphenols, the predominant flavonol and anthocyanin being kaempferol 3-*O*-sophoroside and delphinidin 3,5-di-*O*-glucoside, respectively. Future work should focus on the stability of SPW extracts in DES, as well as on the bioactivity of the extracts, to fully evaluate their potency as food and cosmetic ingredients and pharmaceutical formulations.

## Figures and Tables

**Table 1:** Codified and actual values of the independent variables considered for the experimental design.

Independent Variables	Code Units	Coded Variable Level		
		−1	−1	−1
*C*_DES_ (%, *w*/*v*)	X_1_	55	70	85
R_L/S_ (mL g^−1^)	X_2_	20	40	60
S_S_ (rpm)	X_3_	200	500	800

**Table 2:** Extraction yields and antioxidant characteristics of the saffron processing waste (SPW) extracts obtained with DES and the control solvents.

Solvent	Y_TP_ (mg GAE g^−1^ dm)	Y_TFn_ (mg RtE g^−1^ dm)	A_AR_ (μmol DPPH g^−1^ dm)	P_R_ (μmol AAE g^−1^ dm)
Water	102.91 ± 2.57	49.77 ± 2.99	284.66 ± 5.69	136.14 ± 2.04
60% EtOH	112.15 ± 2.80	53.98 ± 3.24	290.54 ± 5.81	137.18 ± 2.56
60% MeOH	107.13 ± 2.68	54.86 ± 3.29	300.71 ± 6.01	129.05 ± 2.09
DES	120.50 ± 3.01 *	61.27 ± 3.37	213.05 ± 4.26	144.66 ± 3.07

* Asterisk indicates statistically different value (*p* < 0.05).

**Table 3:** Statistical data related with the model established by implementing response surface methodology.

Term	Standard Error	*t* Ratio	Probability > *t*	Sum of Squares	*F* Ratio
*C*_DES_	1.237502	2.62	0.0472 *	83.98080	6.8548
R_L/S_	1.237502	7.01	0.0009 *	601.17781	49.0705
S_S_	1.237502	4.63	0.0057 *	262.54861	21.4303
*C*_DES_ R_L/S_	1.750093	−2.92	0.0329 *	104.65290	8.5422
*C*_DES_ S_S_	1.750093	−2.81	0.0374 *	97.02250	7.9194
R_L/S_ S_S_	1.750093	−1.90	0.1165	44.02322	3.5934
*C*_DES_ *C*_DES_	1.821554	−1.56	0.1793	29.84188	2.4358
R_L/S_ R_L/S_	1.821554	0.64	0.5475	5.09408	0.4158
S_S_ S_S_	1.821554	0.49	0.6441	2.95488	0.2412

* Asterisk indicates statistically different value (*p* < 0.05).

**Table 4:** Measured and predicted values of the response for every point of the experimental design implemented.

Design Point	Independent Variables			Response (Y_TP_, mg GAE g^−1^ dm)	
	X_1_ (*C*_DES_, % *w*/*v*)	X_2_ (R_L/S_, mL g^−1^)	X_3_ (S_S_, rpm)	Measured	Predicted
1	−1 (55)	−1 (20)	0 (500)	93.36	96.63
2	−1 (55)	1 (60)	0 (500)	122.14	124.20
3	1 (85)	−1 (20)	0 (500)	115.40	113.34
4	1 (85)	1 (60)	0 (500)	123.72	120.45
5	0 (70)	−1 (20)	−1 (200)	100.43	99.68
6	0 (70)	−1 (20)	1 (800)	118.23	117.77
7	0 (70)	1 (60)	−1 (200)	123.19	123.65
8	0 (70)	1 (60)	1 (800)	127.72	128.47
9	−1 (55)	0 (40)	−1 (200)	102.00	99.48
10	1 (85)	0 (40)	−1 (200)	113.00	115.81
11	−1 (55)	0 (40)	1 (800)	123.60	120.79
12	1 (85)	0 (40)	1 (800)	114.90	117.42
13	0 (70)	0 (40)	0 (500)	116.25	115.32
14	0 (70)	0 (40)	0 (500)	115.00	115.32
15	0 (70)	0 (40)	0 (500)	114.72	115.32

**Table 5:** Ultraviolet-visual and mass spectrometric data of the major polyphenols detected in the DES extracts of SPW, obtained under optimal conditions.

No	Rt (min)	UV-vis	[M + H]^+^ (m/z)	Fragment Ions (m/z)	Tentative Identity
*Flavonols*					
1	21.08	265, 346	773	611, 449, 287	Kaempferol 3-*O*-sophoroside 7-*O*-glucoside
2	29.81	254, 351	627	303	Quercetin 3-*O*-sophoroside
3	32.63	265, 346	611	449, 287	Kaempferol 3-*O*-sophoroside
4	38.87	265, 352	449	287	Kaempferol 3-*O*-glucoside
*Anthocyanins*					
5	15.62	274, 523	627	465	Delphinidin 3,5-di-*O*-glucoside
6	18.72	272, 523	641	465	Petunidin 3,5-di-*O*-glucoside
7	20.08	271, 523	465	303	Delphinidin 3-*O*-glucoside

**Table 6:** Quantitative values of the major polyphenols detected in the SPW, obtained with the DES under optimal conditions.

Polyphenol	Content (mg g^−1^ dm) ± sd
*Flavonols*	
Kaempferol 3-*O*-sophoroside 7-*O*-glucoside	3.92 ± 0.27
Quercetin 3-*O*-sophoroside	3.55 ± 0.25
Kaempferol 3-*O*-sophoroside	36.43 ± 2.55
Kaempferol 3-*O*-glucoside	1.82 ± 0.13
*Total*	*45.72*
*Anthocyanins*	
Delphinidin 3,5-di-*O*-glucoside	6.28 ± 0.44
Petunidin 3,5-di-*O*-glucoside	1.08 ± 0.08
Delphinidin 3-*O*-glucoside	0.70 ± 0.05
*Total*	*8.06*
*Sum*	*53.79*

## Nomenclature

A_AR_	antiradical activity (μmol DPPH g^−1^)
P_R_	reducing power (μmol AAE g^−1^)
R_L/S_	liquid-to-solid ratio (mL g^−1^)
*t*	time (min)
*T*	temperature (°C)
Y_TFn_	yield in total flavonoids (mg RtE g^−1^)
Y_TP_	yield in total polyphenols (mg GAE g^−1^)
**Abbreviations**	
AAE	ascorbic acid equivalents
DES	deep eutectic solvents
DPPH	2,2-diphenyl-1-picrylhydrazyl radical
GAE	gallic acid equivalents
HBA	hydrogen bond acceptor
HBD	hydrogen bond donor
TPTZ	2,4,6-tripyridyl-*s*-triazine
